# Neoadjuvant Pembrolizumab and Chemotherapy in Resectable Esophageal Cancer: An Open-Label, Single-Arm Study (PEN-ICE)

**DOI:** 10.3389/fimmu.2022.849984

**Published:** 2022-06-02

**Authors:** Hongtao Duan, Changjian Shao, Minghong Pan, Honggang Liu, Xiaoping Dong, Yong Zhang, Liping Tong, Yingtong Feng, Yuanyuan Wang, Lu Wang, Neil B. Newman, Inderpal S. Sarkaria, John V. Reynolds, Francesco De Cobelli, Zhiqiang Ma, Tao Jiang, Xiaolong Yan

**Affiliations:** ^1^ Department of Thoracic Surgery, Tangdu Hospital, Air Force Medical University, Xi’an, China; ^2^ Department of Pathology, Tangdu Hospital, Air Force Medical University, Xi’an, China; ^3^ Department of Pathology, Xijing Hospital, Air Force Medical University, Xi’an, China; ^4^ Department of Radiation Oncology, Vanderbilt-Ingram Cancer Center, Vanderbilt University Medical Center, Nashville, TN, United States; ^5^ Department of Cardiothoracic Surgery, The University of Pittsburgh School of Medicine and the University of Pittsburgh Medical Center, Pittsburgh, PA, United States; ^6^ Department of Surgery, Trinity Centre, St. James’s Hospital, Dublin, Ireland; ^7^ Department of Radiology, IRCCS San Raffaele Scientific Institute, Milan, Italy; ^8^ Department of Medical Oncology, Senior Department of Oncology, The Fifth Medical Center of PLA General Hospital, Beijing, China

**Keywords:** Pembrolizumab, chemotherapy, resectable esophageal cancer, efficacy, safety, pathological complete response (pCR), major pathological response (MPR)

## Abstract

**Background:**

In this single-arm study, the efficacy and safety of neoadjuvant pembrolizumab plus chemotherapy were evaluated in patients with resectable esophageal squamous cell carcinoma (ESCC).

**Methods:**

This study included patients with ESCC of clinical stages II–IVA who underwent surgery within 4 to 6 weeks after completing treatment with pembrolizumab (200 mg) combined with a conventional chemotherapy regimen (3 cycles). The safety and efficacy of this combination treatment were evaluated as primary endpoints of the study.

**Results:**

From April 2019 to August 2020, a total of 18 patients (including 14 men) were enrolled, of whom 13 patients progressed to surgery. Postoperative pathology revealed a major pathological response (MPR) in 9 cases (9/13, 69.2%) and a pathological complete response (pCR) in 6 cases (6/13, 46.2%). Five patients (5/18, 27.8%) experienced serious treatment-related adverse events (AEs) of grades 3–4. At the time of data cutoff (Mar 25, 2022), the shortest duration of follow-up was 17.8 months. Programmed death-ligand 1 (PD-L1) expression in pretreatment specimens was not significantly associated with the percentage of residual viable tumor (RVT) (r=−0.55, P=0.08). Changes in counts of CD68^+^ macrophage between pre- and post-treatment specimens were weakly correlated with RVT (r=0.71; P=0.07), while a positive correlation was observed between postoperative forkhead box P3-positive (Foxp3)^+^T cells/CD4^+^Tcells ratios and RVT (r=0.84, P*=*0.03).

**Conclusions:**

The combination of neoadjuvant immunotherapy and chemotherapy for ESCC is associated with a high pathological response and immunologic effects in the tumor microenvironment (TME). It has acceptable toxicity and great efficacy, suggesting a strong rationale for its further evaluation in randomized clinical trials (RCTs).

**Trial Registration:**

ChiCTR2100048917.

## 1 Introduction

Esophageal cancer (EC) is the 7th most common cancer-related death globally. In China, it is the 6_th_most common malignancy, with esophageal squamous cell carcinoma (ESCC) being the dominant subtype (1). The median overall survival (OS) of patients with advanced or metastatic EC is extremely poor. For patients who have undergone surgeries alone, OS rates are improving; nonetheless, the five-year survival rate does not exceed 50% (2).

According to the current National Comprehensive Cancer Network (NCCN) guidelines, multimodal therapy with neoadjuvant chemoradiotherapy is the recommended standard therapy for patients with T2-4aNxM0 resectable ESCC. The CROSS Study is the definitive modern randomized clinical trial (RCT), with an OS of 48.6 versus 24.0 months in the multimodal and surgery-only cohorts, respectively (3). However, a major limitation for this treatment may be a heightened risk of major respiratory complications (including pneumonia, acute respiratory distress syndrome, respiratory failure, and pulmonary embolism) and mortality postoperatively (4). Consequently, with the advent of a greater understanding of EC tumor biology and genomics, novel approaches that combine efficacy and safety are being explored.

In this regard, there is currently enormous interest in therapies that target the immune cells within the tumor microenvironment (TME). Programmed cell death protein-1 (PD-1) inhibitors have been evaluated in multiple clinical trials. In the KEYNOTE-181 study, pembrolizumab vs. chemotherapy was evaluated as a second-line treatment for advanced (unresectable or metastatic) EC. For patients with PD-L1 combined positive score (CPS) ≥10, the 12-month OS rate was 43% in the pembrolizumab group and 20% in the chemotherapy group (5). And in the KEYNOTE-590 trial, the combination of pembrolizumab and chemotherapy vs. chemotherapy was evaluated as a first-line treatment for the unresectable or metastatic EC. The survival rate at 12 months of ESCC was higher with chemoimmunotherapy versus chemotherapy (51% vs. 38%) (6). In the neoadjuvant treatment of non-small cell lung cancer (NSCLC), PD-1 inhibitors have produced excellent results. In the NCT02716038 study, NADIM study, and our recent trial, neoadjuvant chemoimmunotherapy in resectable NSCLC reported encouraging data of pathological response, with MPR (57%, 83%, 50% respectively) and pCR (33%, 63%, 30% respectively) (7–9).

Given these encouraging trends for neoadjuvant regimens including anti-PD-1 therapy, the present study aimed to explore the safety and efficacy of anti-PD-1 therapy combined with chemotherapy for resectable ESCC in neoadjuvant settings. We also preliminarily explore the correlations between pathological response and immunological parameters of the TME.

## 2 Methods

### 2.1 Patients

Patients gave their informed consent to participate in the study, and the study was approved by the Ethics Committee of Tangdu Hospital of the Fourth Military Medical University (approval No. 202005-12-KY-07-XW-01).

In this single-arm study, 18 patients were enrolled. The eligibility criteria were: (I) aged 18 years or older; (II) Eastern Cooperative Oncology Group (ECOG) score 0-1; (III) histologically confirmed ESCC of clinical stages II–IVA (T2-4aNxM0; for theT2N0M0, the tumor length should be ≥2 cm under endoscopy; When the tumor is located in the cervical segment, the tumor boundary should be more than 5 cm away from the annular pharyngeal muscle. Lymph node with a short diameter ≥10 mm is considered as metastatic lymph node) [according to the American Joint Committee on Cancer (AJCC 8th edition)]; (IV) surgically resectable ESCC evaluated by a multidisciplinary clinical team; (V) normal hematologic, renal, and hepatic function; (VI) receiving guidance on contraception if necessary; (VII) obtained written consent inform. The exclusion criteria were: (I) patients with active autoimmune disease; (II) patients with active concurrent malignancy; (III) patients receiving ongoing systemic steroids (>10 mg daily prednisone equivalents); (IV) patients having received radiotherapy, target therapy, chemotherapy, or other immunosuppressive therapy; (V) patients severe allergic to pembrolizumab, its active substance and/or any excipients (grade ≥3); (VI) patients severely allergic to the investigational chemotherapeutic drug and/or any of its excipients (grade ≥3); (VII) history of HIV positive testing or known acquired immune deficiency syndrome; (VIII) history of hepatitis B or active hepatitis C virus infection; (IX) history of active tuberculosis; (X) pregnant women; (XI) women in the lactation state.

### 2.2 Procedures

All patients were arranged with pretreatment staging. It consists of upper gastrointestinal endoscopy with histological biopsy, computed tomography (CT) scan of the chest, ultrasonography of liver, pancreas, spleen, kidney and cervical lymph nodes, pulmonary function test, echocardiography and radionuclide bone scintigraphy. For patients with suspected cervical lymph node involved, external ultrasonography of the neck with fine-needle aspiration or positron emission tomography-CT (PET-CT) was required.

All of the included patients were scheduled to receive the following drugs intravenously: pembrolizumab (200 mg) combined with conventional chemotherapy for three 21-day cycles prior to surgical resection. Chemotherapy regimens were referred to platinum-based two-drug combination chemotherapy. And the specific chemotherapeutic regimen of each patient was performed under investigators’ choices. The detailed regimens were shown in **Table 1**.

**Table 1:** All detailed data about patients.

Patient No.	Sex	Age (years)	Location of tumor	Clinical TNM (cTNM)	Chemotherapy	RECIST 1.1	Post-Neoadjuvant therapy TNM (ypTNM)	pCR/MPR	RVT%	PD-L1 CPS	Number of lymph nodes resected
P1	F	59	Middle	cT3N1M0 Stage III	Docetaxel (75m/m^2^, D1) +Nedaplatin (80m/m^2^, D1)	Non-CR/non-PD	ypT0N0M0 Stage I	pCR	0		22
P2	M	66	Middle	cT3N1M0 Stage III	Nab-paclitaxel (260 mg/m²,D1) +Nedaplatin (80m/m^2^, D1)	Non-CR/non-PD	ypT3N1M0 Stage IIIB		20	30	48
P3	M	54	Middle	cT4N1M0 Stage IVA	Docetaxel (75m/m^2^, D1) +Nedaplatin (80m/m^2^, D1)	SD	ypT4N0M0 Stage IIIB		70	<1	25
P4	M	56	Middle	cT3N2M0 Stage III	Docetaxel (75m/m^2^, D1) +Nedaplatin (80m/m^2^, D1)	PR	ypT3N2M0 Stage IIIB		60	15	17
P5	M	35	Distal	cT3N1M0 Stage III	Nab-paclitaxel (260 mg/m²,D1) +Nedaplatin (80m/m^2^, D1)	PR	ypT0N0M0 Stage I	pCR	0	45	16
P6	F	51	Middle	cT3N1M0 Stage III	Docetaxel (75m/m^2^, D1) +Nedaplatin (80m/m^2^, D1)	Non-CR/non-PD	ypT4N0M0 Stage IIIB		65	<1	48
P7	M	66	Middle	cT3N2M0 Stage III	Nab-paclitaxel (260 mg/m²,D1) +Nedaplatin (80m/m^2^, D1)	PR	YpT1bN0M0 Stage I	MPR	9	10	25
P8	M	59	Middle	cT3N0M0 Stage II	Nab-paclitaxel (260 mg/m²,D1) +Nedaplatin (80m/m^2^, D1)	CR	ypT0N0M0 Stage I	pCR	0		15
P9	M	59	Middle	cT3N0M0 Stage II	Nab-paclitaxel (260 mg/m²,D1) +Nedaplatin (80m/m^2^, D1)	Non-CR/non-PD	ypT2N0M0 Stage I	MPR	≤10		33
P10	M	66	Middle	cT3N1M0 Stage III	Nab-paclitaxel (260 mg/m²,D1) +Nedaplatin (80m/m^2^, D1)	PR	ypT0N0M0 Stage I	pCR	0	20	26
P11	M	64	Middle	cT3N1M0 Stage III	Nab-paclitaxel (130 mg/m²,D1,8) +Nedaplatin (80m/m^2^, D1)	Non-CR/non-PD	ypT0N1M0 Stage IIIA	MPR	≤10		26
P12	F	65	Middle	cT3N0M0 Stage II	Nab-paclitaxel (130 mg/m²,D1,8) +Nedaplatin (80m/m^2^, D1)	CR	ypT0N0M0 Stage I	pCR	0	16	29
P13	F	57	Middle	cT3N0M0 Stage II	Nab-paclitaxel (130 mg/m²,D1,8) +Nedaplatin (80m/m^2^, D1)	CR	ypT0N0M0 Stage I	pCR	0	6	27
P14	M	78	Middle	cT3N1M0 Stage III	Nab-paclitaxel (130 mg/m²,D1,8) +Nedaplatin (80m/m^2^, D1)	Non-CR/non-PD					
P15	M	67	Middle	cT3N0M0 Stage II	Docetaxel (75m/m^2^, D1) +Nedaplatin (80m/m^2^, D1)	CR					
P16	M	71	Distal	cT3N1M0 Stage III	Nab-paclitaxel (130 mg/m²,D1,8) +Nedaplatin (80m/m^2^, D1)	PR				<1	
P17	M	67	Distal	cT3N0M0 Stage II	Nab-paclitaxel (130 mg/m²,D1,8) +Nedaplatin (80m/m^2^, D1)	Non-CR/non-PD					
P18	M	67	Middle	cT3N0M0 Stage II	Nab-paclitaxel (260 mg/m²,D1) +Nedaplatin (80m/m^2^, D1)	PD				<1	

pCR, pathological complete response; MPR, major pathological response; RVT, residual viable tumor; PD-L1, programmed death-ligand 1; CPS, combined positive score; CR, complete response; PD, progressive disease; SD, stable disease; PR, partial response.

After the neoadjuvant therapy was completed, reevaluation was performed to exclude patients with contraindication to surgery. The reevaluation included tests the same as pretreatment staging. Surgery was planned within 4–6 weeks after the completion of the induction regimen. McKeown or IvorLewis esophagectomy, including two-field lymphadenectomy with total mediastinal lymph node dissection, was performed according to standard institutional procedures. For patients with cervical lymph nodes involved, three-field lymph node dissection was required.

### 2.3 Experimental Design

The primary endpoints were safety and efficacy.

#### 2.3.1 Safety

Adverse events (AEs) were assessed according to the Common Terminology Criteria for Adverse Events (CTCAE) V.4.0.

#### 2.3.2 Efficacy

Efficacy was measured according to the following criteria: (I) pathological complete response (pCR), defined as the complete absence of tumor cells (corresponds with MANDARD TRG 1 and Becker grade 1a), or major pathological response (MPR), defined as <10% residual viable tumor (RVT) (it is generally corresponds with MANDARD treatment response grade(TRG) 1 and 2, or Becker pathologic response grade 1a + 1b), or incomplete pathological response, defined as ≥10% RVT (non-MPR/non-pCR) (10); (II) symptom remission, according to the Stooler classification (11); (III) treatment radiographic response, as determined using the Response Evaluation Criteria in Solid Tumors (RECIST version1.1). According to RECIST version1.1, target lesions of EC were defined as lymph nodes with a short diameter ≥15 mm, and primary esophageal lesions were not considered as target lesions. When lesions were consistent with the standards stated above, the definitions of complete response (CR), partial response (PR), stable disease (SD) and progressive disease (PD)were consistent with the standards stated in RECIST 1.1; (IV)R0 resection was defined as circumferential resection margin (CRM) greater than 1 mm and proximal - distal resection margins are free.

### 2.4 Evaluation of Immunologic Parameters

#### 2.4.1 Clinical Samples

Upper gastrointestinal endoscopy with histological biopsy was carried out to obtain the pre-treatment specimens, and post-operative specimens were collected from surgical excisions. All samples were prepared for follow-up testing.

#### 2.4.2 Immunohistochemistry

PD-L1 expression was detected in pre-treatment formalin-fixed, paraffin-embedded tumor samples using IHC. Batch assays were performed on all samples using the PD-L1 IHC SP263 pharmDx assay according to the manufacturer’s instructions. The combined positive score (CPS) were evaluated by two pathologists as described previously.

#### 2.4.3 Immune Signature of Clinical Samples

Forkhead box P3-positive (Foxp3), CD4-positive and CD8-positive tumor-infiltrating lymphocytes were compared between pre-treatment biopsy specimens and surgical specimens using multiplex immunofluorescence (mIF) (Shanghai Baili Biotechnology Co. Ltd., Shanghai, China). CD68-positive macrophages and the expression of programmed death-ligand 1 (PD-L1), tumor necrosis factor-alpha (TNF)-α, and transforming growth factor-beta 1 (TGF)-β1 were also analyzed by immunohistochemistry (online Supplementary Methods). The Foxp3^+^T cells/(CD4+T cells) ratios and Foxp3^+^T cells/(CD8+T cells) ratios were calculated.

### 2.5 Statistical Analysis

Demographic and safety data, as well as clinical, pathologic, radiographic, and molecular response data, were recorded using descriptive statistics. The associations between RVT and pretreatment PD-L1 expression were analyzed using Spearman’s correlation analysis. Furthermore, the associations of RVT and other pretreatment and posttreatment immune parameters combined with their changes were analyzed using Spearman’s correlation analysis, including the expression of TNF-α, TGF-β1, and the counts of Foxp3^+^ CD4^+^, CD8^+^ T cells and CD68^+^ macrophages. Similarly, the associations of RVT and pretreatment and posttreatment Foxp3^+^T cells/(CD4^+^T cells) ratios and Foxp3^+^T cells/(CD8^+^T cells) ratios combined with their changes were analyzed using Spearman’s correlation analysis. Additionally, the differences in pCR between patients whose PD-L1 CPS (≥10) and CPS <10 were analyzed with χ² test. All P values reported are 2-sided, with the significance level set at 0.05. Statistical analyses were performed using SPSS 19.0.

## 3 Results

### 3.1 Baseline Demographic and Clinical Characteristics

**Figure 1:**
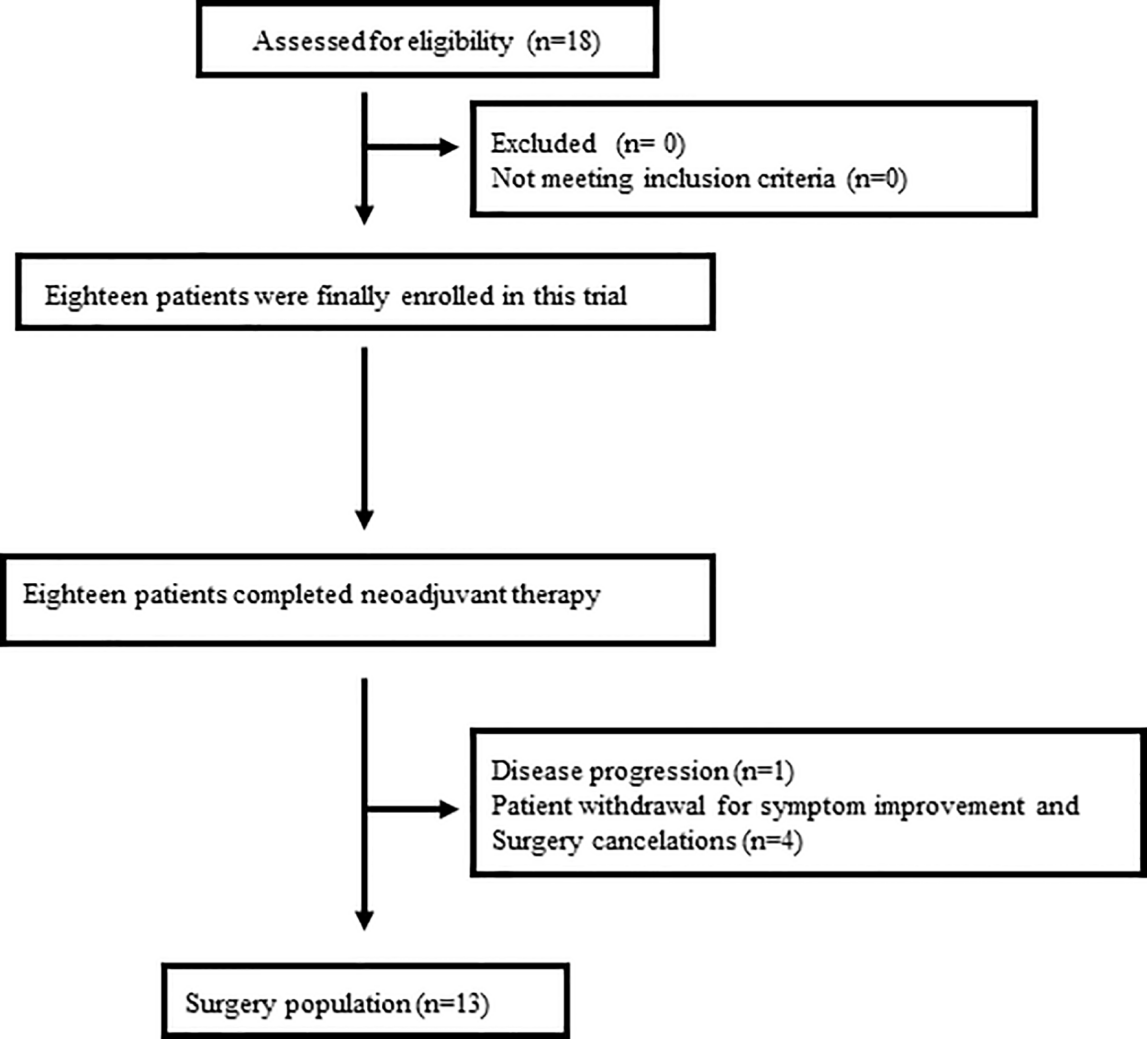
Patients enrolled.

From April 2019 to August 2020, 18 patients were enrolled in this study (**Figure 1**), of which the median age was 64 years old and the average age was 60.9 years (ranged from 35 to 78 years). Males accounted for 77.8% (14 patients) of the patients while females accounted for 22.2% (4 patients). The locations of tumors were the middle third of esophagus (15/18, 83.3%) and the distal third of esophagus (3/18, 16.7%). Before the scheduled therapy, 7 patients (38.9%) were classified into clinical tumor stage II, 10 patients (55.6%) into stage III, and 1 patients (5.6%) into stage IV. The detailed data of patients were summarized in **Table 1**.

### 3.2 Efficacy

**Figure 2:**
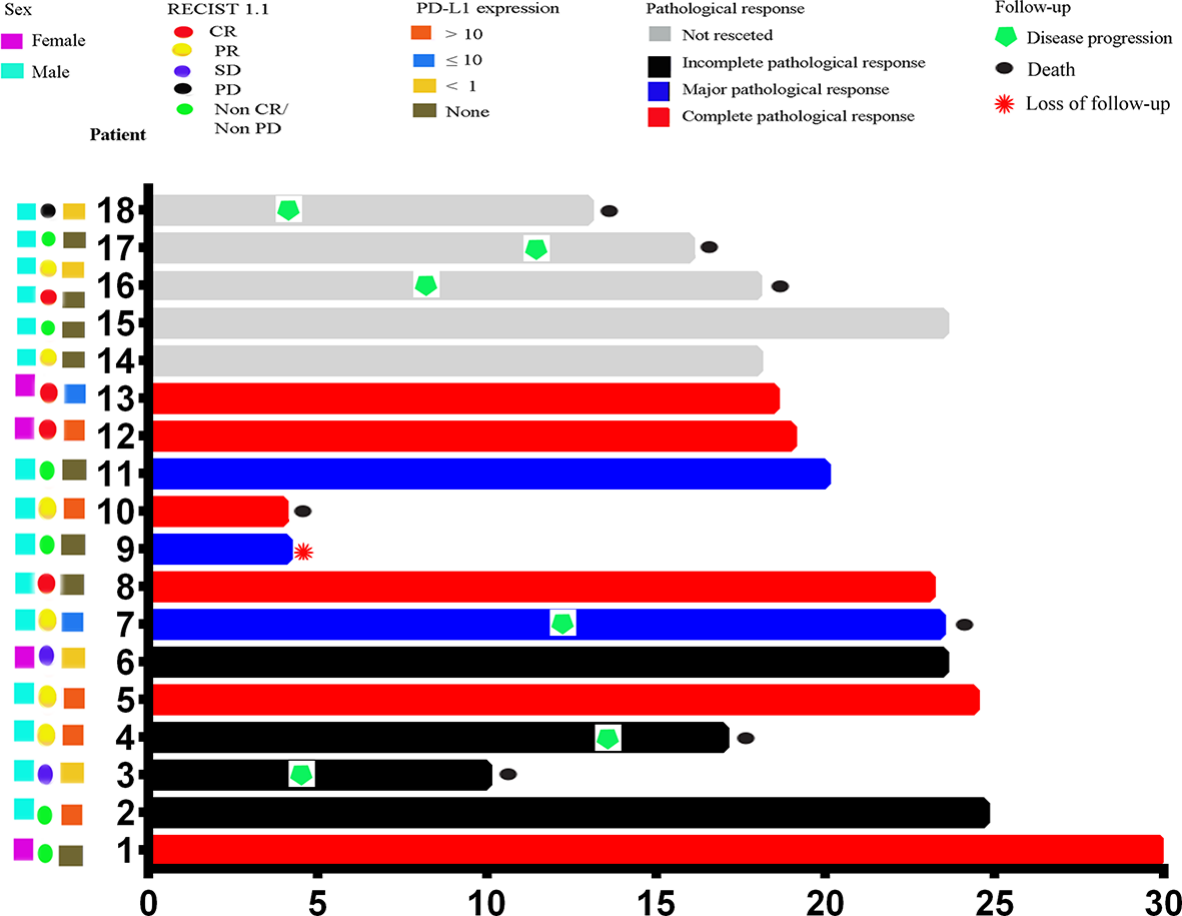
Swimmer plot of PFS in the modified intention-to-treat population (n = 18). Each bar represents one patient. The left column shows clinical characteristics. PFS, progression-free survival.

**Figure 3:**
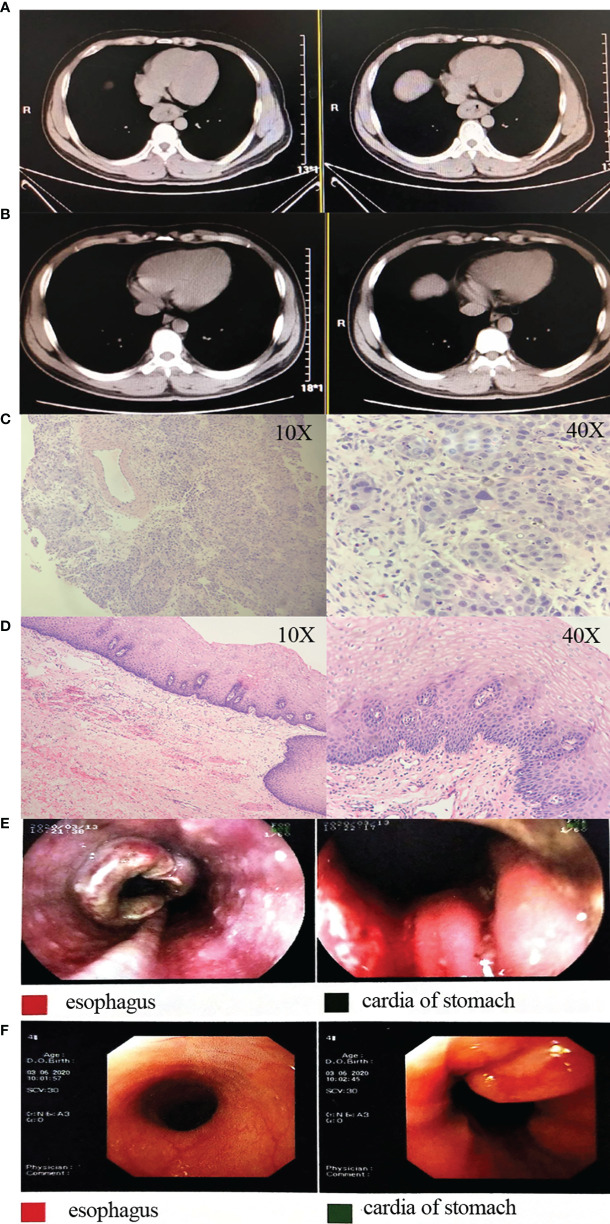
Representative images and pathology for a patient. **(A)** Pre-treatment CT images; **(B)** post-treatment CT images; **(C)** pre-treatment HE staining images; **(D)** postoperative HE staining images; **(E)** pre-treatment gastroscopy images; **(F)** post-treatment gastroscopy images. CT, computed tomography; HE, hematoxylin and eosin.

This study enrolled 18 patients, of whom 13 patients progressed to surgery, 4 patients refused surgery due to significant tumor regression and symptomatic relief, and 1 patient experienced disease progression (patient 18) and was given definitive chemoradiotherapy (**Figure 1**). The pathological stages of the patients who underwent surgery after treatment were as follows: stage I (8 patients), stage IIIA (1 patients), and stage IIIB (4 patients). Tumor downstaging occurred in 69.2% (9/13) of patients. In this study, R0 resection was defined as circumferential resection margin (CRM) greater than 1 mm. Therefore, R0 resection rate was 84.6% (11/13), R1 resection rate 7.7% (1/13) and R2 resection rate 7.7% (1/13). The mean number of lymph nodes resected during operation was 27.5 (range, 15–48). Postoperative pathological response revealed a pCR in 6 cases (46.2%), and an MPR in 9 cases (69.2%). **Figure 1** shows representative imaging and pathology of a patient with pCR. According to the Stooler classification, before neoadjuvant treatment, 2 patients (22.7%) had stenosis of grade 3 and 16 patients (77.3%) had stenosis of grade 2. While after therapy, 16 patients (88.9%) had grade 0 symptoms, and 2 patients (11.1%) had grade 2 symptoms, with a symptom remission rate of 94.4%. According to RECIST 1.1, 5 patients (27.8%) had a PR, and 4 patients (22.2%) attained a CR, which translated to an objective response rate (ORR) of 50% (**Figure 2**). Intriguingly, in patients achieving MPR, the size of primary lesions was all decreased and the rate of symptom remission was 100%. All detailed data were shown in **Table 1**. Representative imaging and pathology for a patient with PCR was shown in **Figure 3**.

### 3.3 Survival

**Figure 4:**
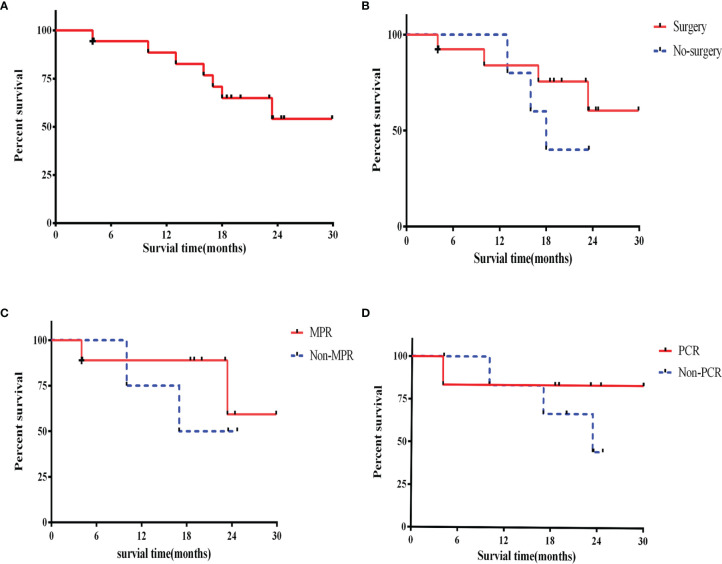
Overall survival. **(A)** Overall survival in the intention-to-treat population; **(B)** overall survival for surgery patients and no surgery; **(C)** overall survival for MPR and non-MPR patients; **(D)** overall survival for pCR and non-pCR patients. +, censoring. MPR, major pathological response; pCR, pathological complete response.

Except for loss of follow-up of one patient, all patients had a good follow-up record. At the time of data cutoff (Mar 25, 2022), the shortest duration of follow-up was 17.8 months. and the median follow-up time of the survivors is 23.0 (17.8–29.9) months. Kaplan-Meier analysis for OS was shown in **Figure 4A**. The median OS was 16.0 months at the non-surgery group while the surgical group has not yet reached the median OS (**Figure 4B**).

In addition, the OS rates in MPR and pCR group were slightly higher than that in non-MPR [hazard ratio (HR) =0.49; 95% confidence interval (CI): 0.06–3.96; P=0.40] and non-pCR group (HR =0.43; 95% CI: 0.06–3.14; P=0.62), respectively (**Figures 4C, D**).

### 3.4 Safety and Surgical Complications

AEs are shown in **Table 2**. None of the patients in the study discontinued treatment due to an AE. The most common treatment-related AEs of grade 1 or 2 were leukopenia, neutropenia, anorexia, vomiting, fatigue, and alopecia. Five patients (5/18, 27.8%) experienced serious treatment-related AEs of grade 3–4 (including anemia, neutropenia, anorexia, vomiting, fatigue, and alopecia). Hypothyroidism, skin rashes and pneumonitis were attributed as possibly being related to the immunotherapy.

**Table 2:** Treatment-related adverse events.

Variables	Any grade	Grade 1–2	Grade 3	Grade 4
Anemia	2	2	1	
Leukopenia	7	7		
Neutropenia	7	7	1	
Immune thrombocytopenic purpura	1	1		
Anorexia	8	7	1	
Vomiting	9	8	1	
Diarrhea	2	2		
Fatigue	10	9	1	
Alopecia	6	4	2	
Hypothyroidism	1	1		
Skin rashes	2	2		
Pneumonitis	1	1		

Thirteen patients received surgery. Postoperative complications included hoarseness (5 cases, 38.%), pneumonia (4 cases, 30.8%), empyema (3 cases, 23.1%), atelectasis (2 cases, 15.4%), heart failure (2 cases, 15.4%), respiratory failure (1 case, 7.7%), and anastomotic leak (1 case, 7.7%). Patient 11 developed pneumonitis and pneumonia on postoperative day 1, after treatment with antimicrobial drugs and steroids (2 mg/kg), and his conditions gradually worsened until his death on postoperative day 22.

### 3.5 Pathologic Assessment and Genomic Analyses

Samples from 11 patients before treatment (pre-treatment) and 7 patients after three cycles of neoadjuvant immunochemotherapy (post-treatment) were obtained. Samples at both time points were available for 7 patients (patients 2, 3, 4, 5, 6, 7, 10).

**Figure 5:**
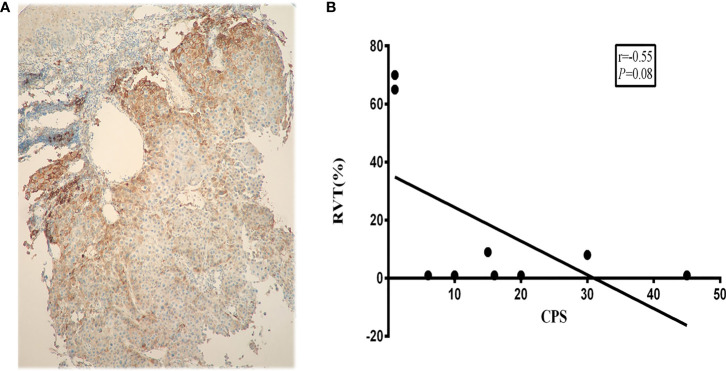
Correlation between PD-L1 expression and RVT. **(A)** Representative PD-L1 IHC image of pre-treatment specimens; **(B)** correlation analysis of PD-L1 expression and RVT. PD-L1, programmed death-ligand 1; RVT, residual viable tumor; IHC, immunohistochemistry.

The data suggested that RVT was not significantly associated with pre-treatment PD-L1 expression (r=−0.55; P=0.08) (**Figure 5**). Additionally, between patients whose PD-L1 CPS (≥10) and CPS <10, no significant differences in pCR were identified.

**Figure 6:**
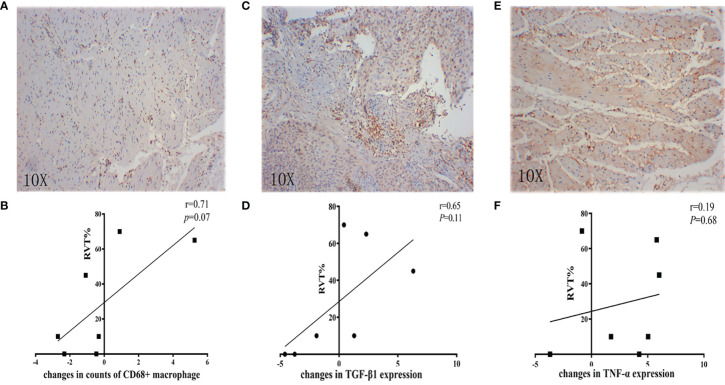
Correlation between inflammatory parameters and RVT. **(A, C, E)** Representative IHC image of CD68, TGF-β1, and TNF-α; **(B)** correlation analysis between changes in counts of CD68^+^ macrophage and RVT; **(D)** correlation analysis between changes in TGF-β1 expression and RVT; **(F)** correlation analysis between changes in TNF-α expression and RVT. RVT, residual viable tumor; IHC, immunohistochemistry; TGF-β1, transforming growth factor-beta 1; TNF-α, tumor necrosis factor-alpha.

**Figure 7:**
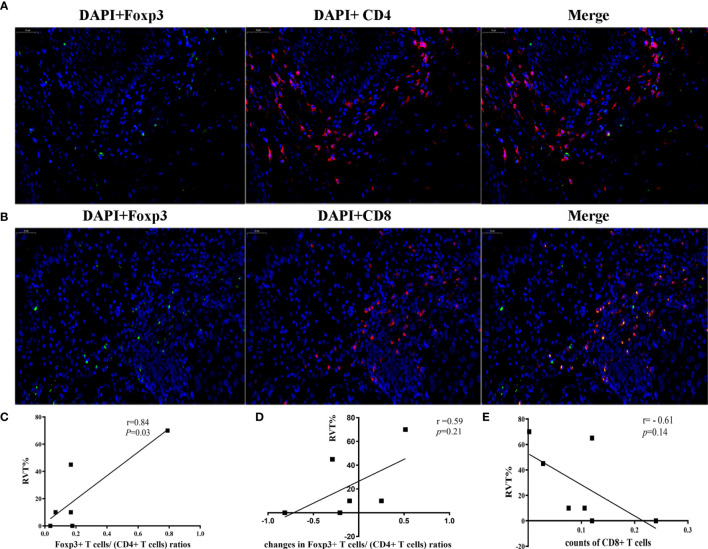
Correlation between immune cells and RVT. **(A)** Two-color immunofluorescence analysis showing the expression of CD4, Foxp3. DAPI (blue), CD4 (red) and Foxp3 (green); **(B)** Two-color Immunofluorescence analysis showing the expression of CD8, Foxp3. DAPI (blue), CD8 (red) and Foxp3 (green); **(C)** correlation analysis between postoperative Foxp3^+^ T cells/(CD4^+^ T cells) ratios and RVT; **(D)** correlation analysis between changes in Foxp3^+^ T cells/(CD4^+^ T cells) ratios and RVT; **(E)** correlation analysis between the counts of postoperative CD8^+^ T cells and RVT. RVT, residual viable tumor; DAPI, DAPI (4’,6-diamidino-2-phenylindole) fluorescence marking the cell nucleus.

Further analysis showed that the changes in counts of CD68^+^ macrophage were found to be positively correlated with RVT (r = 0.71; P = 0.07) (**Figures 6A, B**). To explore the relationship between inflammatory cytokines and RVT, immunohistochemical methods were adopted to examine pre- and post-treatment expression of TNF-α and TGF-β1 in the pathologic specimens (**Figures 6C–F**). In this study, the post-treatment expression of TGF-β1 was increased compared to the preoperative expression, and the changes in TGF-β1 expression were positively correlated with RVT (r=0.65, P=0.11) and possibly indicated a poor prognosis (**Figures 6C, D**). However, the available data showed no significant correlation between the changes in TNF-α expression and RVT (**Figures 6E, F**). The correlations of RVT and the parameters of lymphocyte populations stated above were further explored (**Figures 7A, B**), with a positive correlation observed between postoperative Foxp3^+^ T cells/(CD4^+^ T cells) ratios and RVT (r=0.84, P*=*0.03) (**Figure 7C**), positive correlation observed between changes in Foxp3^+^ T cells/(CD4^+^ T cells) ratios and RVT (r=0.59, P=0.21) (**Figure 7D**) and negative correlation observed between the counts of postoperative CD8^+^ T cells and RVT (r = −0.61, P=0.14) (**Figure 6E**). However, no direct correlation was found between other immune cells and RVT.

## 4 Discussion

Different from PALACE-1, which combines immunotherapy and chemoradiotherapy for resectable EC in neoadjuvant settings, our study is the first to report on pembrolizumab combined with chemotherapy alone in the neoadjuvant treatment of EC (12). Our study was done in our hospital involving 18 patients, small sample size though, important observations were made. At the same time, our trail is the first to report neoadjuvant chemoimmunotherapy for ESCC about OS, except for loss of follow-up of one patient (4 months) and the death of patient (4.1 months), and the longest or shortest follow-up time was 29.9 or 17.8 months. The OS rates of 1-year is similar to previous studies (3, 13).

Firstly, the pCR of 46.2% was high, not dissimilar to the rates observed with chemoradiation in the CROSS trial (in ESCC, 49%) and the NEOCRTEC 5010 RCT (43.2%) (3, 13). We want to show that two patients who had moderate-severe atypical hyperplasis after surgery were identified as having pCR. This level of pathological response has not been observed in previous studies with chemotherapy alone in neoadjuvant settings, of which the pCR was typically less than 20% (12.8%) (14). Furthermore, in a recent retrospective study that performed two cycles of combination of chemotherapy and immunotherapy in neoadjuvant settings of ESCC, their results revealed a pCR of 22.2% to 35.3%, lower than the pCR in our study. It’s supposed that the cycles of neoadjuvant treatment may affect the efficacy, which deserves further studies to be confirmed (15, 16). Moreover, pCR and MPR are verified to confer a survival advantage and to prolong median disease-free survival (DFS) in EC and many other cancers (10). To validate the DFS and OS benefits of neoadjuvant chemoimmunotherapy for ESCC, further exploration of this regimen could be done in this patient population. Additionally, in this study, for patients with no target-lesions, the standards of RICIST1.1 system merely consist of CR, PD, non-CR/non-PD, which makes it difficult to obtain the values of ORR. Therefore, the accuracy of RICIST1.1 system in evaluating the efficacy of neoadjuvant immunochemotherapy in ESCC needs further exploration.

We also evaluated the safety of the regimen. In our study, only 5 of 18 patients (27.8%) experienced treatment-related AEs of grade 3 or 4. The incidence of serious AEs appears to be acceptable, compared to the PALACE-1 study (65%) and NADIM study (30%) (8, 12). Furthermore, treatment with neoadjuvant pembrolizumab did not delay planned surgery. Immune-related AEs, both hypothyroidism and hypoadrenalism, were identified and relieved with supplementary treatment, with no delay to surgery. The single postoperative death (1/13; 7.7%) which had been diagnosed pulmonary fibrosis (Grade 1) at a high risk of pneumonitis was diagosed to pneumonitis and pneumonia, suggesting that we need additional attention to the treatment of complications in such patients (17).

The associations between RVT and the immunologic parameters are intriguing. In the TME, tumor cells, blood vessels, immune cells, lymphocytes, cancer stem cells, and cancer-associated fibroblasts mix, and considerable immune cell activity may be stage- and context-dependent (18). Macrophages are a key component of the TME. As the principal cells of antigen recognition and presentation, they secrete TNF-α, interleukin-1β, and other cytokines, and impact the magnitude and type of T-cell response. Studies report that a high macrophage count was associated with poor OS (19), and this conclusion may also account for the positive correlation between RVT and changed CD68 expression in the post-treatment pathologic tissues compared with the pre-treatment samples. Accordingly, CD68 expression may be predictive of a poor response to immunotherapy, and this requires further study. Immunosuppressive cells [i.e., regulatory T cells (Tregs, Foxp3^+^ T)] are a part of infiltrating CD4^+^T-cell in the TME, which significantly inhibit the T-cell-mediated anti-tumor effect and may be associated with T-cell dysfunction (20, 21). In other translational studies, Foxp3^+^ T cells in the TME of NSCLC were associated with poor OS (22), and in our study, a positive correlation between post-treatment Foxp3^+^ T cells/(CD4^+^ T cells) ratios and RVT was confirmed in the context of ESCC, suggesting a prognostic role of post-treatment Foxp3^+^ T cells/(CD4^+^ T cells) ratios. No direct correlation was found between the counts of other T cells and RVT. These results may be attributable to the time and space heterogeneity of immunotherapy, although the specific mechanism requires further study. TGF-β1, TNF-α and other cytokines in the TME also play key roles in regulating the response to immunotherapy (23). Among these cytokines, immunoregulatory TGF-β1 suppresses the proliferation of B-cell, cytotoxic T-cell, and natural killer cell and antagonizes the biological effects of TNF-α (24). In other studies, it was reported that TGF-β signaling may counteract anti-tumor immunity by restricting the movement of T-cells in the TME (25). We hypothesized that the increases in TGF-β may predict poor pathological response. This hypothesis was supported by our findings that changes in TGF-β1 expression were positively correlated with RVT. It may be difficult that the small sample size in this study precluded a full analysis of the relationship between TNF-α, TGF-β1, and RVT, and further study is required.

There are some limitations to this study that should be noted. Firstly, other markers of relevance including the genomic profile, tumor mutational burden, and the inflammatory factor interferon-gamma were not evaluated. A further limitation is the study’s small sample size; however, since it is the first clinical trial of its kind, it still has important clinical observational significance to serve as the backbone for larger analyses. Another important aspect is that the OS of patients was not extensively explored, longer follow-up time would be used. Mostly, this is an open-label, single-arm study, so there are bias in enrollment.

## 5 Conclusions

In conclusion, neoadjuvant chemoimmunotherapy is safe and feasible for patients with ESCC, with an extremely high pCR and MPR and a clear impact on the TME. With adjuvant studies (such as Checkmate 577 trial) of anti-PD-1 therapy in EC having reported promising results, further RCTs and translational studies should be performed with this treatment paradigm, which appears to hold considerable potential.

## Data Availability Statement

The original contributions presented in the study are included in the article/supplementary material. Further inquiries can be directed to the corresponding authors.

## Ethics Statement

The studies involving human participants were reviewed and approved by the Ethics Committee of Tangdu Hospital of the Fourth Military Medical University. The patients/participants provided their written informed consent to participate in this study. Written informed consent was obtained from the individual(s) for the publication of any potentially identifiable images or data included in this article.

## Author Contributions

Conception and design: XY, TJ and ZM. Administrative support: XY and TJ. Provision of study materials or patients: HD, HL, XD, YZ and LT. Collection and assembly of data: HD, CS, MP, HL, XD, YZ, LT and YF. Data analysis and interpretation: LT, YW, LW, NN, IS, JR and FDC. Manuscript writing: All authors. Final approval of manuscript: All authors. All authors contributed to the article and approved the submitted version.

## Funding

The present study was supported by grants from the National Natural Science Foundation of China (No. 81871866) and AIRC Investigator Grant 2019 (No.23015).

## Conflict of Interest

The authors declare that the research was conducted in the absence of any commercial or financial relationships that could be construed as a potential conflict of interest.

## Publisher’s Note

All claims expressed in this article are solely those of the authors and do not necessarily represent those of their affiliated organizations, or those of the publisher, the editors and the reviewers. Any product that may be evaluated in this article, or claim that may be made by its manufacturer, is not guaranteed or endorsed by the publisher.
